# Analgesic Effects and the Mechanisms of Anti-Inflammation of Hispolon in Mice

**DOI:** 10.1093/ecam/nep027

**Published:** 2010-10-25

**Authors:** Heng-Yuan Chang, Ming-Jyh Sheu, Chun-Hung Yang, Tsung-Chun Lu, Yuan Shiun Chang, Wen-Huang Peng, Shyh-Shyun Huang, Guan-Jhong Huang

**Affiliations:** ^1^Institute of Chinese Pharmaceutical Sciences, Taichung 40402, Taiwan; ^2^School of Pharmacy, College of Pharmacy, China Medical University, Taichung 404, Taiwan; ^3^Department of Pesticide Toxicology, Taiwan Agricultural Chemicals and Toxic substances Research institute, Wu Feng, Taichung 433, Taiwan

## Abstract

Hispolon, an active ingredient in the fungi *Phellinus linteus* was evaluated with analgesic and anti-inflammatory effects. Treatment of male ICR mice with hispolon (10 and 20 mg/kg) significantly inhibited the numbers of acetic acid-induced writhing response. Also, our result showed that hispolon (20 mg/kg) significantly inhibited the formalin-induced pain in the later phase (*P<*.01). In the anti-inflammatory test, hispolon (20 mg/kg) decreased the paw edema at the fourth and fifth hour after *λ*-carrageenin (Carr) administration, and increased the activities of superoxide dismutase (SOD), glutathione peroxidase (GPx) and glutathione reductase (GRx) in the liver tissue. We also demonstrated that hispolon significantly attenuated the malondialdehyde (MDA) level in the edema paw at the fifth hour after Carr injection. Hispolon (10 and 20 mg/kg) decreased the nitric oxide (NO) levels on both the edema paw and serum level at the fifth hour after Carr injection. Also, hispolon (10 and 20 mg/kg) diminished the serum TNF-*α* at the fifth hour after Carr injection. The anti-inflammatory mechanisms of hispolon might be related to the decrease in the level of MDA in the edema paw by increasing the activities of SOD, GPx and GRx in the liver. It probably exerts anti-inflammatory effects through the suppression of TNF-*α* and NO.

## 1. Introduction

In Asia, macrofungi are commonly given as a nutritional supplement to patients with a variety of diseases [1]. Several different species of *Phellinus* are believed to have anticancer as well as antioxidant properties. *Phellinus linteus* (PL) has been demonstrated to exhibit anti-tumor and antioxidant properties [2]. The methanolic extract of the basidiocarps of PL exhibits an antioxidative effect [3]. Studies have indicated that PL could protect primary cultured rat hepatocytes against hepatotoxins [4], and *P. rimosus* (Berk) Pilat seems to exhibit antioxidant and antihepatotoxic activities [5]. There is also a report indicating that *n*-BuOH subfraction of PL exhibited anti-inflammatory activity [6]. Hispolon, an active ingredient in the fungi *P. igniarius* [7] has been reported to induce apoptosis in human epidermoid KB cells [8]. Hispolon has also been shown to inhibit the chemiluminescence response of human mononuclear cells and suppress mitogen-induced proliferation of spleen lymphocytes in mice [9]. In our previous study, we found that ethanol extract of *P. merrillii* (PM) displayed antioxidant activities in a series of *invitro* tests [10]. PM also showed hepatoprotective and antioxidant effects in Sprague-Dawley rat with carbon-tetrachloride-induced liver damage [11]. However, little information is available on the analgesic and anti-inflammatory effects of hispolon.

Some researches demonstrate that inflammatory effect induced by Carr could be associated with free radical. Free radical, prostaglandin and nitric oxide (NO) were released when Carr was administered for 1–6 h [12]. The edema effect was raised to maximum at the third hour [13] and its MDA production was due to free-radical attack on plasma membrane [14]. Thus, inflammatory effect would result in the accumulation of MDA. Therefore, in this article we examined the analgesic effects of hispolon on nociception induced by acetic acid and formalin. We also evaluated the anti-inflammatory effects of hispolon on paw edema induced by Carr in mice. Furthermore, we detected the levels of MDA, NO and TNF-*α* in either paw edema or serum. Also, the activities of SOD, GPx and GRx in the liver at the fifth hour after Carr injection were investigated to understand the relationship between the anti-inflammatory mechanism of hispolon and antioxidant enzymes.

## 2. Materials and Methods

### 2.1. Chemicals

**Figure 1:**
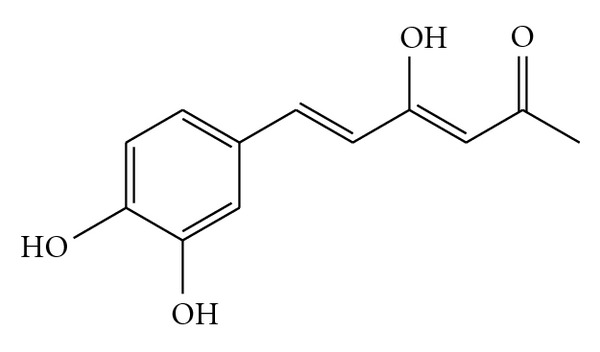
Chemical structure of hispolon.

Hispolon (Figure 1) was synthesized as described and its purity was established on the basis of the spectra (^1^H, ^13^C NMR and mass) data [15]. Acetic acid was purchased from Merck (Darmstadt, Germany). Carr and indomethacin were obtained from Sigma (St Louis, MO, USA). Formalin was purchased from Nihon Shiyaku Industries (Japan). TNF-*α* was purchased from Biosource International Inc. (Camarillo, CA, USA).

### 2.2. Animals

Six- to eight-week-old male ICR mice were obtained from the BioLASCO Taiwan Co., Ltd. The animals were kept in plexiglass cages at a constant temperature of 22 ± 1°C, relative humidity 55 ± 5% with 12 h dark–light cycle for at least 2 weeks before the experiment. They were given food and water *ad libitum*. All experimental procedures were performed according to the NIH Guide for the Care and Use of Laboratory Animals. The placebo groups were given 0.1 ml/10 g saline intraperitoneally using a bent blunted 27-gauge needle connected to a 1 mL syringe. All tests were conducted under the guidelines of the International Association for the Study of Pain [16].

After a 2-week adaptation period, male ICR mice (18–25 g) were randomly assigned to five groups (*n* = 8) in acetic-acid-induced writhing and formalin-induced licking groups. These include a normal (received acetic acid or formalin), a positive control (acetic acid or formalin + indomethacin (Indo)) and hispolon administered groups (acetic acid or formalin + hispolon). In the Carr-induced edema experiment, there were six groups (*n* = 8) of the animals in the study. The control group received normal saline. The other five groups included a Carr-treated, a positive control (Carr + Indo) and hispolon-administered groups (Carr + hispolon).

### 2.3. Acetic-Acid-Induced Writhing Response

The test was performed as described by Fontenele et al. [17]. Nociception was induced by an intraperitoneal (i.p.) injection of 0.1 mL/10 g acetic acid solution (10 mL/kg). Positive control animals were pretreated with Indo (10 mg/kg, i.p.) 25 min before acetic acid. Each hispolon administered group was pretreated with 5 mg/kg, 10 mg/kg or 20 mg/kg i.p. 25 min before acetic acid. Five minutes after the i.p. injection of acetic acid, the number of writhing and stretching was recorded.

### 2.4. Formalin Test

The antinociceptive activity of the drugs was determined using the formalin test described by Dubuisson and Dennis [18]. Twenty microliters of 5% formalin was injected into the dorsal surface of the right hind paw of mice 30 min after i.p. administration of the hispolon (5, 10 and 20 mg/kg), Indo. The mice were observed for 30 min after the injection of formalin, and the amount of time spent licking the injected hind paw was recorded. The first 5 min post formalin injection is referred to as the early phase and the period between 15 min and 40 min as the late phase. The total time spent licking or biting the injured paw (pain behavior) was measured with a stop watch. The activity was recorded in 5 min intervals.

### 2.5. *λ*-Carrageenin-Induced Edema

Carr-induced hind paw edema model was used for determination of anti-inflammatory activity [19]. Animals were i.p. treated with the hispolon (5, 10 and 20 mg/kg), Indo or normal control, 30 min prior to injection of 1% Carr (50 *μ*l) in the plantar side of right hind paws of the mice. Paw volume was measured immediately after Carr injection and at 1, 2, 3, 4 and 5 h intervals after the administration of the edematogenic agent using a plethysmometer (model 7159, Ugo Basile, Varese, Italy). The degree of swelling induced was evaluated by the ratio *a*/*b*, where *a* is the volume of the right hind paw after Carr treatment, and *b* is the volume of the right hind paw before Carr treatment. Indo was used as a positive control. After 5 h, the animals were sacrificed, the Carr-induced edema feet were dissected and stored at −80°C. Blood samples were withdrawn and kept at −80°C.

#### 2.5.1. Total Protein Assay

The protein concentration of the sample was determined by the Bradford dye-binding assay (Bio-Rad, Hercules, CA).

#### 2.5.2. MDA Assay

MDA from Carr-induced edema foot was evaluated by the thiobarbituric acid reacting substance (TRARS) method [20]. Briefly, MDA reacted with thiobarbituric acid in the acidic high temperature and formed a red-complex TBARS. The absorbance of TBARS was determined at 532 nm.

#### 2.5.3. Determination of NO

NO determinations were carried out in 100 *μ*L aliquots of sample mixed with 100 *μ*L of the Griess reagent [21]. Nitrite was quantified by using sodium nitrate as a standard curve.

#### 2.5.4. Measurement of Serum TNF-*α* by ELISA

Serum levels of TNF-*α* were determined using a commercially available enzyme linked immunosorbent assay (ELISA) kit according to the manufacturer's instruction. TNF-*α* was determined from a standard curve for the combination of these cytokines. The concentrations were expressed as pg/mL [22].

#### 2.5.5. Antioxidant Enzymes Activity Measurements

Liver tissue homogenates were collected for the estimation of SOD [23], GPx [24] and GRx enzyme [25] to detect the antioxidant activities of hispolon.

#### 2.5.6. Histological Examination

For histological examination, biopsies of paws were taken 5 h following the interplanetary injection of Carr. The tissue slices were fixed in (1.85% formaldehyde, 1% acetic acid) for 1 week at room temperature, dehydrated by graded ethanol and embedded in Paraffin (Sherwood Medical). Sections (thickness 5 *μ*m) were deparaffinized with xylene and stained with H & E stain. All samples were observed and photographed with BH2 Olympus microscopy. Every three to five tissue slices were randomly chosen from Carr-, Indo- and hispolon-treated (20 mg/kg) groups. The numbers of neutrophils were counted in each scope (400x) and thereafter obtain their average count from five scopes of every tissue slice.

#### 2.5.7. Statistical Analysis

Data are expressed as mean ± SEM. Statistical evaluation was carried out by one-way analysis of variance (ANOVA followed by Scheffe's multiple range test). Statistical significance is expressed as *P <  .05, **P <  .01, ***P <  .001.

## 3. Results

### 3.1. Effects of Hispolon on Acetic-Acid-Induced Writhing Response

**Figure 2:**
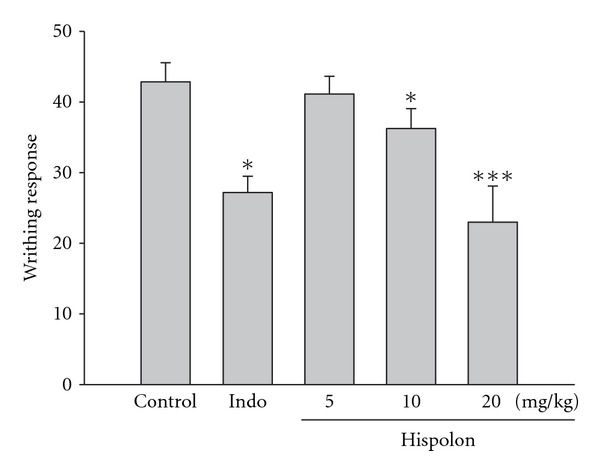
Analagesic effects of the hispolon and indomethacin (Indo) on acetic-acid-induced writhing response in mice. Each value represents as mean ± SEM. *P <  .05 and ***P <  .001 as compared with the control (Con) group (one-way ANOVA followed by Scheffe's multiple range test).

The cumulative amount of abdominal stretching correlated with the level of acetic-acid-induced pain (Figure 2). Hispolon treatment (10 mg/kg) significantly inhibited the number of writhing in comparison with the normal controls (*P* <  .05). Hispolon (20 mg/kg) further inhibited the number of writhing (*P* <  .001), and it demonstrates more inhibition than that produced by Indo (10 mg/kg).

### 3.2. Formalin Test

**Figure 3:**
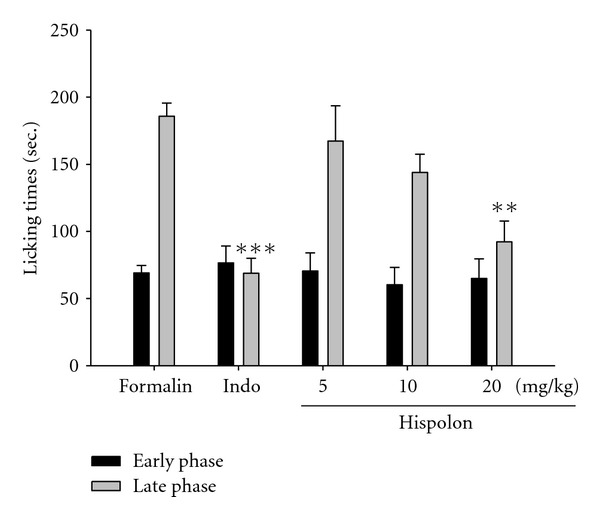
Effects of the hispolon and indomethacin (Indo) on the early phase and late phase in formalin test in mice. Each value represents as mean ± SEM. **P <  .01 and ***P <  .001 as compared with the control (Con) group (one-way ANOVA followed by Scheffe's multiple range test).

Hispolon (20 mg/kg) significantly (*P* <  .01) inhibited formalin-induced pain in the late phase (Figure 3); however, it did not show any inhibition in the early phase. The positive control Indo (10 mg/kg) also significantly (*P* <  .001) inhibited the formalin-induced pain in the late phase.

### 3.3. Effects of Hispolon on *λ*-Carrageenan-Induced Mice Paw Edema

**Figure 4:**
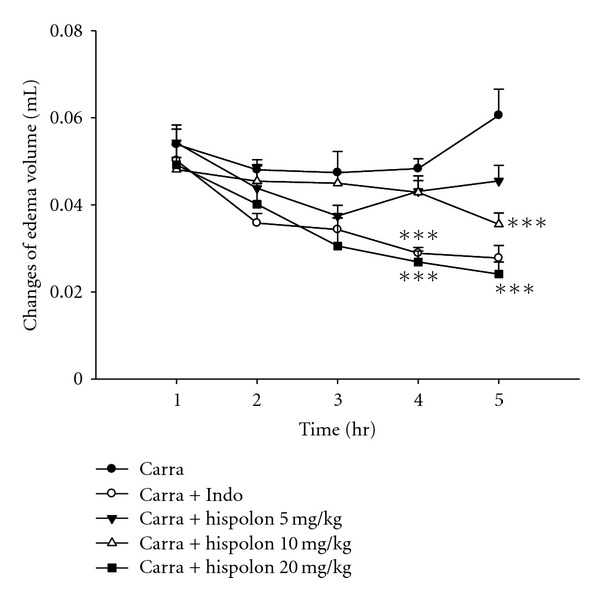
Effects of the hispolon and indomethacin (Indo) on hind paw edema induced by *λ*-carrageenan (Carr) in mice. Each value represents as mean ± SEM. ***P <  .001 as compared with the Carr group (one-way ANOVA followed by Scheffe's multiple range test).

As shown in Figure 4, Carr induced paw edema. Hispolon (20 mg/kg) significantly inhibited (*P* <  .001) the development of paw edema induced by Carr after 4 and 5 h of treatment. Indo (10 mg/kg) significantly decreased the Carr-induced paw edema after 4 and 5 h of treatment (*P* <  .001).

#### 3.3.1. Effects of Hispolon on MDA Level Measurements

**Figure 5:**
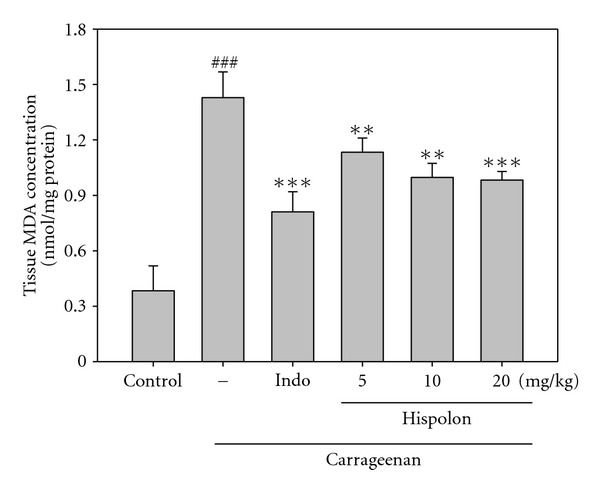
Effects of the hispolon and indomethacin (Indo) on the tissue MDA concentration of foot in mice. Each value represents as mean ± SEM. ^###^ *P* <  .001 as compared with the control group. **P <  .01 and ***P <  .001 as compared with the Carr group (one-way ANOVA followed by Scheffe's multiple range test).

MDA level increased significantly in the edema paw at the 5 h after Carr injection (*P* <  .001). However, MDA level was decreased significantly by treatment with hispolon (5, 10 and 20 mg/kg) (*P* <  .001 or *P*<  .001), as well as 10 mg/kg Indo (Figure 5).

#### 3.3.2. Effects of Hispolon on NO Measurement

**Figure 6:**
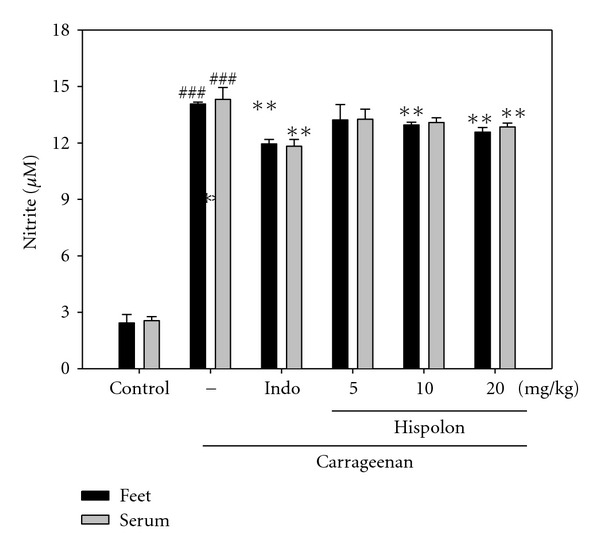
Effects of the hispolon and indomethacin (Indo) on carrageenan (Carr)-induced NO concentration of edema paw and serum at fifth hour in mice. Each value represents as mean ± SEM. ^###^ *P* <  .001 as compared with the control group. *P <  .05, **P <  .01 and ***P <  .001 as compared with the Carr group (one-way ANOVA followed by Scheffe's multiple range test).

In Figure 6, the NO level increased significantly in the edema paw and serum at the 5 h after Carr injection (*P* <  .001). Hispolon (10 and 20 mg/kg) significantly decreased the serum NO level (*P* <  .05 or *P* <  .01). The inhibitory potency was similar to that of Indo (10 mg/kg) at 5 h after induction.

#### 3.3.3. Effects of Hispolon on TNF-*α* Measurement

**Figure 7:**
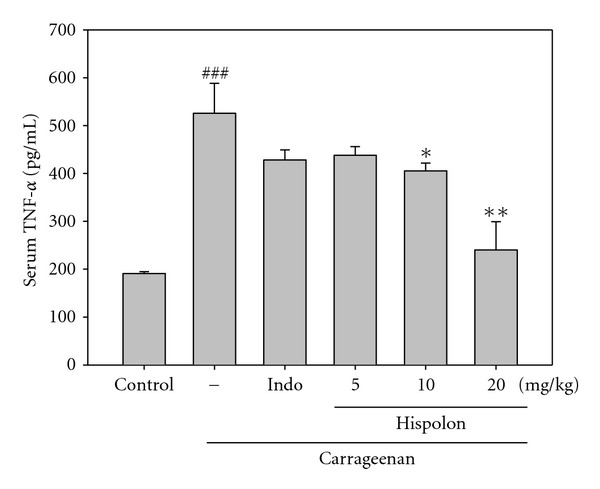
Effects of the hispolon and indomethacin (Indo) on carrageenan (Carr)-induced NO concentration of serum at fifth hour in mice. Each value represents as mean ± S.E.M. ^###^ *P* <  .001 as compared with the control group. *P <  .05 and **P <  .01 as compared with the Carr group (one-way ANOVA followed by Scheffe's multiple range test).

TNF-*α* level increased significantly in serum at the 5 h after Carr injection (*P* <  .001). However, hispolon (10 and 20 mg/kg) decreased the TNF-*α* level in serum at the 5 h after Carr injection (*P* <  .05 or *P* <  .01), as well as 10 mg/kg Indo (Figure 7).

#### 3.3.4. Effects of Hispolon on Activities of Antioxidant Enzymes

At the fifth hour following the intrapaw injection of Carr, liver tissues were also analyzed for the biochemical parameters such as SOD, GPx and GRx activities (Table 1). SOD activity in liver tissue was decreased significantly by Carr administration. SOD activity was increased significantly after treated with 20 mg/kg hispolon and 10 mg/kg Indo (*P* <  .01) (Table 1). Carr administration markedly decreased GPx and GRx activities in the livers. GPx and GRx activities of the livers were increased significantly by hispolon (10 and 20 mg/kg), as well as Indo (10 mg/kg) (Table 1).

#### 3.3.5. Histological Examination

**Figure 8:**
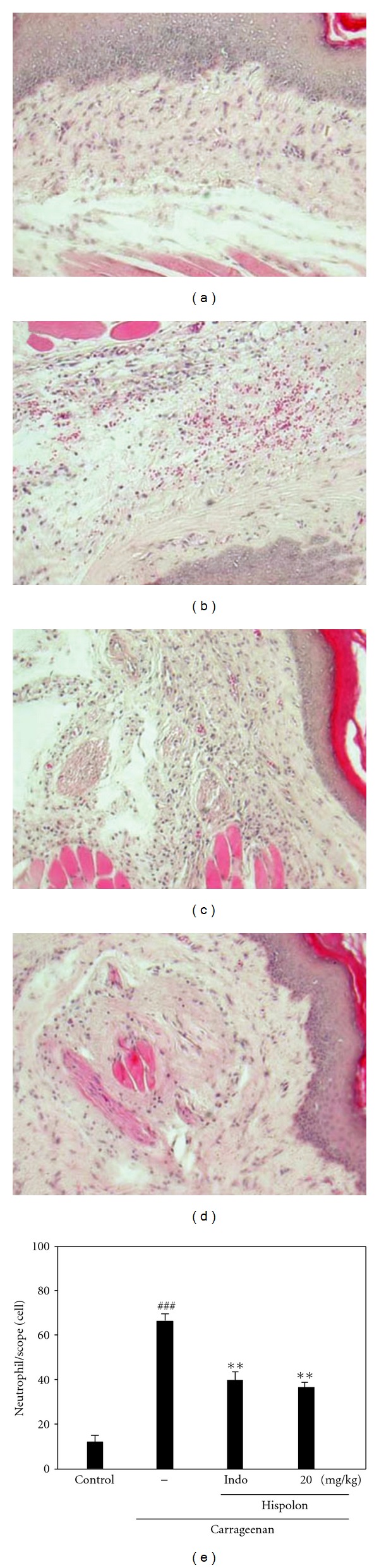
Histological appearance of the mouse hind footpad after a subcutaneous injection with 0.9% saline (control group) or carrageenan (Carr) stained with H&E stain. (a). Control rats: show the normal appearance of dermis and subdermis without any significantly lesion. (b). Hemorrhage with moderately extravascular red blood cell and large amount of inflammatory leukocyte mainly neutrophils infiltration in the subdermis interstitial tissue of mice following the subcutaneous injection of Carr only. Moreover, detail of the subdermis layer show enlargement of the interstitial space caused by edema with exudates fluid. (c). Indomethacin (Indo) significantly reduced the level of hemorrhage, edema and inflammatory cell infiltration compared to subcutaneous injection of Carr only. (d). Hispolon significantly show morphological alterations compared to subcutaneous injection of Carr only. (100x) (e). The numbers of neutrophils were counted in each scope (400x) and thereafter obtain their average count from five scopes of every tissue slice. **P <  .01, compared with Carr group.

Paw biopsies of control animals showed marked cellular infiltration in the connective tissue. The infiltrates accumulated between collagen fibers and into intercellular spaces. Paw biopsies of animals treated with hispolon (20 mg/kg) showed a reduction in Carr-induced inflammatory response. Actually inflammatory cells were reduced in number and confined near to the vascular areas. Intercellular spaces did not show any cellular infiltrations. Collagen fibers were regular in shape and showed a reduction of intercellular spaces. Moreover, the hypoderm connective tissue was not damaged (Figure 8). Neutrophils were increased with Carr treatment (*P* <  .001). Indo and hispolon (20 mg/kg) could significantly decrease the neutrophils numbers as compared to the Carr-treated group (*P* <  .01) (Figure 8(e)).

## 4. Discussion

We have evaluated the putative analgesic and anti-inflammatory activities of hispolon to clarify the pain and inflammation relieving effects. Two different analgesic testing methods were employed with the objective of identifying possible peripheral and central effects of the test substances. The acetic-acid writhing test is normally used to study the peripheral analgesic effects of drugs. Although this test is non-specific (e.g. anticholinergic, antihistaminic and other agents also show activity in the test), it is widely used for analgesic screening [26]. In our study, we found that hispolon (10 and 20 mg/kg) exhibited antinociceptive effect in acetic-acid-induced writhing response (Figure 2). This effect may be due to inhibition of the synthesis of the arachidonic acid metabolites [27].

The *invivo* model of pain, formalin-induced paw pain has been well established as a valid model for analgesic study. The formalin test produces a distinct biphasic response and different analgesics may act differently in the early and late phases of this test. Therefore, the test can be used to clarify the possible mechanism of an antinociceptive effect of a proposed analgesic [28]. Centrally acting drugs such as opioids inhibit both phases equally [26], but peripherally acting drugs such as aspirin, Indo and dexamethasone only inhibit the late phase. The inhibitory effect of hispolon on the nociceptive response in the late phase of the formalin test suggested that the anti-nociceptive effect of the hispolon could be due to its peripheral action (Figure 3).

The Carr test is highly sensitive to non-steroidal anti-inflammatory drugs, and has long been accepted as a useful phlogistic tool for investigating new drug therapies [29]. The degree of swelling of the Carr-injected paws was maximal 3 h after injection. Statistical analysis revealed that hispolon and Indo significantly inhibited the development of edema 4 h after treatment (*P* <  .001) (Figure 4). They both showed anti-inflammatory effects in Carr-induced mice edema paw. It is well known that the third phase of the edema-induced by Carr, in which the edema reaches its highest volume, is characterized by the presence of prostaglandins and other compounds of slow reaction [30]. It was found that the injection of Carr into the rat paw induces the liberation of bradykinin, which later induces the biosynthesis of prostaglandin and other autacoids, which are responsible for the formation of the inflammatory exudates. In addition, the classification of antinociceptive drugs is usually based on their mechanism of action either on the central nervous system or on the peripheral nervous system [31].

In the studies of mechanism on the inflammation, l-arginine-NO pathway has been proposed to play an important role in the Carr-induced inflammatory response [32]. Our present results also confirm that Carr-induced paw edema model results in the production of NO. The expression of the inducible isoform of NO synthase has been proposed as an important mediator of inflammation [33]. In our study, the level of NO was decreased significantly by treatment with 10 and 20 mg/kg hispolon. We suggest the mechanism of anti-inflammatory of hispolon may be through the l-arginine-NO pathway since hispolon significantly inhibit the NO production (Figure 6).

TNF-*α* is a major mediator in inflammatory responses, inducing innate immune responses by activating T cells and macrophages, and stimulating secretion of other inflammatory cytokines [34]. Also, TNF-*α* is a mediator of Carr-induced inflammatory incapacitation, and is able to induce the further release of kinins and leukotrienes, which is suggested to have an important role in the maintenance of long-lasting nociceptive response [35]. In this study, we found hispolon decreased the TNF-*α* level in serum after Carr injection (Figure 7).

The Carr-induced inflammatory response has been linked to neutrophils infiltration and the production of neutrophils-derived free radicals, such as hydrogen peroxide, superoxide and hydroxyl radicals, as well as the release of other neutrophils-derived mediators [36]. Some researches demonstrate that inflammatory effect induced by Carr is associated with free radical. Free radical, prostaglandin and NO will be released when administrating with Carr for 1–6 h [12]. The edema effect was raised to the maximum at the third hour [13]. Janero demonstrated that MDA production is due to free-radical attack on plasma membrane [37, 38]. Thus, inflammatory effect would result in the accumulation of MDA. Glutathione is a known oxyradical scavenger. Enhances the level of Glutathione conducive toward favor reduces MDA the production. Cuzzocrea [39] suggested that endogenous glutathione plays an important role against Carr-induced local inflammation. In this study, significantly increase in SOD, GRx and GPx activities with hispolon treatment was found (Table 1). Furthermore, there were significantly decreases in MDA level with hispolon treatment (Figure 5). We assume that the suppression of MDA production is probably due to the increase of SOD, GRx and GPx activities.

In conclusion, these results suggested that hispolon possessed analgesic and anti-inflammatory effects. The anti-inflammatory mechanism of hispolon may be related to iNOS and it is associated with the increase in the activities of antioxidant enzymes (SOD, GPx and GRx). Hispolon may be used as a pharmacological agent in the prevention or treatment of disease in which free radical formation is a pathogenic factor.

## Funding

China Medical University (CMU96-113 and CMU97-232); National Science Council (NSC 97-2313-B-039 -001 -MY3).

## Figures and Tables

**Table 1:** Effects of hispolon and indomethacin (Indo) on the liver SOD, GPx and GRx activities in mice.

Groups	SOD (U/mg protein)	GPx (U/mg protein)	GRx (U/mg protein)
Carr	17.84 ± 1.20	0.357 ± 0.035	0.041 ± 0.005
Carr + Indo	22.48 ± 1.16**	0.636 ± 0.063**	0.053 ± 0.002**
Carr + Hispolon (5 mg/kg)	19.34 ± 2.49	0.571 ± 0.021	0.043 ± 0.004
Carr + Hispolon (10 mg/kg)	20.27 ± 1.82	0.576 ± 0.080*	0.044 ± 0.002
Carr + Hispolon (20 mg/kg)	21.45 ± 2.53*	0.579 ± 0.036**	0.047 ± 0.003*

Each value represents as mean  SEM. *P <  .05 and **P <  .01 as compared with the Carr (*λ*-carrageenan) group (one-way ANOVA followed by Scheffe's multiple range test).
